# Meckel’s diverticulum: a case report from the University Hospital Center Yaoundé, Cameroon

**Published:** 2009-10-15

**Authors:** Marc Leroy Guifo, Vincent Siysi Verla, Alain Mefiré Chichom, Aurelien Ndoumbé, Arthur Essomba, Samuel Takongmo

**Affiliations:** 1 Centre Hospitalier Universitaire, Yaoundé Cameroon,; 2 University of Buea, Cameroon,; 3 Central Hospital Yaoundé.

**Keywords:** Meckel’s diverticulum, acute abdomen, difficult diagnosis, Cameroon

## Abstract

To the best of our knowledge there is no reported case of Meckel’s diverticulum (MD) in Cameroon. The prevalence of MD in the general population is 2–3 %. The aim of this paper is to recapitulate the role of this pathology in acute abdomens and abdominal pain of uncertain aetiology in young patients and to review the medical literature.

## Background

Meckel’s diverticulum is considered as one of the most common gastrointestinal malformation with a prevalence rate of 2–3% [1, 2]. Although well known, few or no cases have been reported in Cameroon. Usually discovered incidentally; it is often the cause of acute abdominal emergencies. It may present as intestinal obstruction with volvulus, intussusceptions or peritonitis due to perforation and lower gastrointestinal hemorrhage [3, 4]. The relatively silent clinical course makes the preoperative diagnosis rare and there is controversy about the attitude to adopt in case of incidental discovery [2]. We report the case of a 26-year-old man who had an intestinal obstruction for whom a resection-anastomosis was done without complications. Informed consent was obtained from the patient for publication of this case report and any accompanying images.

## Patient and case report

TJ a 26-year-old male was brought to the emergency department of the University hospital Center of Yaoundé (Cameroon) for acute diffuse abdominal pain of increasing intensity that started two days earlier. He had vomited and had not passed stool or flatulus for several days. On physical examination he looked very ill and had a temperature of 38.9º C. The blood pressure was 100/40 mmHg, pulse 78 pulsations per minute and the respiratory rate was 16 breaths per minute. The abdomen was slightly distended and moved with breathing. There was diffuse tenderness, no abdominal rigidity and rectal examination was painful with no blood on the glove. The hernia orifices were clear of any mass. An x-ray, ultrasound of the abdomen and the biological work-up with full blood count, coagulation profile were ordered. A nasal-gastric tube and a urinary catheter were inserted and an IV line set up for hydration with Ringer’s lactate solution.

**Figure 1:**
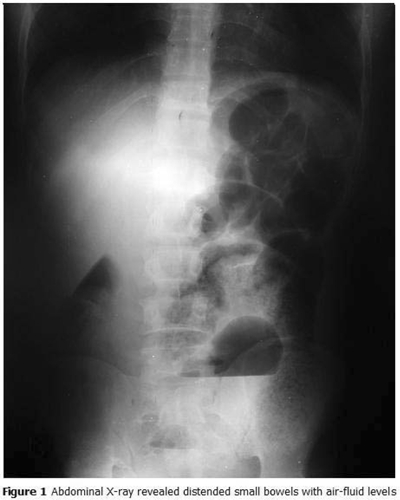
Abdominal X-ray revealed distended small bowels with air-fluid levels

The x-ray revealed distended small bowels with air-fluid levels ( Figure 1 ). Ultrasound showed a distended bowel with no mass or intra-peritoneal fluid. White blood cells count and hemoglobin were 9300/mm ^3^ and 15.6g/dl respectively. Our diagnosis was non-specific intestinal obstruction.

**Figure 2:**
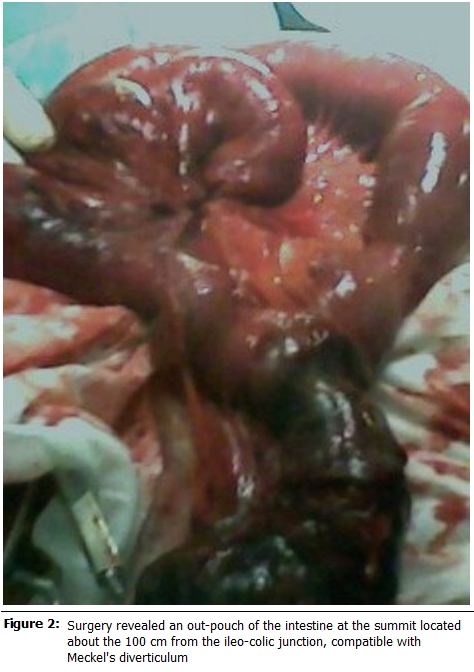
Surgery revealed an out-pouch of the intestine at the summit located about the 100 cm from the ileo-colic junction, compatible with Meckel’s diverticulum

We decided to do an exploratory laparatomy. The findings were a volvulus of the small bowel with a gangrenous portion strangulating the normal looking loops. Upon relieving, we found an out-pouch of the intestine at the summit located about 100cm from the ileo-colic junction, compatible with Meckel’s diverticulum ( Figure 2 ).

The treatment was a resection and an end-to-end anastomosis. The convalescence was uneventful. We did not send the specimen to the pathologist due to the gangrenous nature of the diverticulum.

## Discussion

Although well known before Meckel’s birth in 1781; the persistence of the omphalo-mesenteric duct can give rise to a large spectrum of diseases ranging from the diverticulum of the intestine or the vitelline cyst to the umbilical sinus [5]. Meckel, in 1801 demonstrated the relationship between the diverticulum and the development of the vitelline duct [6]. It is considered the most common congenital anomaly of the gastrointestinal tract [2]. The specific prevalence in Cameroon is difficult to establish due to insufficient report, the classic over 80% benign course in life time and lack of post mortem studies [7]. Most case series report a dozen of patients from individual experience world wide thus the disease is believe to have worldwide distribution. Dietary habits and unknown factors may also contribute to incidence variation in symptomatic cases but this has not been investigated. It may become pathologic, presenting as volvulus, diverticulitis and has heterotopic tissue which may bleed or occasionally become malignant [8]. Intestinal obstruction is the most common presentation of Meckel’s diverticulum [3]. There are no symptoms or physical findings specific for these presentations; therefore any preoperative diagnosis relies on scintigraphy which is not routinelly available in our country. Although very specific (95%), scintigraphy can be falsely positive in enteric duplications, inflammatory bowel disease, haemangioma, intussusception and ectopic kidney [4]. Most of the time it is a peroperative diagnosis or an incidental finding during a laparatomy for reasons not related to the diverticulum. In classical teaching there should be a mandatory search whenever an exploratory laparotomy is inconclusive of appendicitis. There is an unexplained male predilection for the disease with a ratio of 9/5 [9, 10]. The symptomatic cases usually arise in infancy but could still occur at any time as in our case in a 26-year-old man. There is some controversy as to which attitude to adopt since any anastomosis has a potential for complications that should be balanced with the estimated 4% of lifetime complication rate [11]. The host status mainly in the incidental cases should be well studied since infection, malnutrition and immunodeficiency are factors that could seriously affect the outcome of anastomosis. In such cases secondary resection should be considered. There are two ways of managing Meckel’s diverticulum; namely a segment resection with an end-to-end anastomosis and wedge resection. It is believed that the segment resection gives more assurance as heterotopic mucosa removal is concerned [12]. Laparoscopic treatment is another option which provides good results [13].

## Conclusion

From the review of literature and analysis of the above case, the surgeon must always bear in mind the possibility that Meckel’s diverticulum may be the aetiological factor in any acute abdominal emergency, especially when specific diagnosis is difficult. One forty abdominal emergencies may be a MD. A late diagnosis may reflect a long history of abdominal suffering calling for increased pediatric attention.
